# A Systematic Analysis of Narrowband IoT Quality of Service

**DOI:** 10.3390/s20061636

**Published:** 2020-03-14

**Authors:** Andreas Philipp Matz, Jose-Angel Fernandez-Prieto, Joaquin Cañada-Bago, Ulrich Birkel

**Affiliations:** 1 Department of Electrical Engineering and Information Technology, Technische Hochschule Mittelhessen, Wiesenstraße 14, 35390 Gießen, Germany; andreas.p.matz@ei.thm.de (A.P.M.); ulrich.birkel@ei.thm.de (U.B.); 2 Telematic Engineering System Research Group, CEATIC Center of Advanced Studies in Information and Communication Technologies, University of Jaén, Campus Científico-Tecnológico de Linares, C.P. 23700 Linares, Spain; jcbago@ujaen.es

**Keywords:** narrowband IoT, quality of service, performance, mMTC, LPWAN, 5G

## Abstract

Narrowband-IoT (NB-IoT) is part of a novel group of access technologies referred to as Low-Power Wide Area Networks (LPWANs), which provide energy-efficient and long-range network access to IoT devices. Although NB-IoT Release 13 has been deployed by Mobile Network Operators (MNO), detailed Quality of Service (QoS) evaluations in public networks are still rare. In this paper, systematic physical layer measurements are conducted, and the application layer performance is verified. Special consideration is given to the influence of the radio parameters on the application layer QoS. Additionally, NB-IoT is discussed in the context of typical smart metering use cases. The results indicate that NB-IoT meets most theoretical Third Generation Partnership Project (3GPP) design goals in a commercial deployment. NB-IoT provides a wide coverage by using signal repetitions, which improve the receiver sensitivity, but simultaneously increase the system latency. The maximum data rates are consistent over a wide range of coverage situations. Overall, NB-IoT is a reliable and flexible LPWAN technology for sensor applications even under challenging radio conditions. Four smart metering transmission categories are analyzed, and NB-IoT is verified to be appropriate for applications that are not latency sensitive.

## 1. Introduction

In recent years, the rise of the Internet of Things (IoT) and its associated platforms has directed the interest of researchers and companies alike to the exchange of information between IoT endpoints such as sensors and servers. These communication patterns are referred to as Machine Type Communication (MTC) [1]. The communication requirements within the MTC category are diverse; therefore, it is common to distinguish between cMTC (critical MTC) and mMTC (massive MTC) technologies. A widespread mMTC use case is Wireless Sensor Networks (WSNs), which consist of many battery powered devices distributed across heterogeneous environments.

The diverse applications of WSNs have sparked the development of a new group of IoT-centric access technologies. Low-Power Wide Area Networks (LPWANs) provide long-range communication for many devices simultaneously, while enabling battery life for many years. In general, LPWANs can be categorized based on the license of the occupied spectrum. Unlicensed technologies, such as LoRaWAN [2], work in the license-free and region-specific Industrial, Scientific, and Medical (ISM) and Short-Range Devices (SRD) bands. Licensed technologies are standardized by an industry consortium such as the Third Generation Partnership Project (3GPP) [3] and are commonly deployed by Mobile Network Operators (MNOs) along with existing mobile networks. Some LPWANs are developed based on existing standards, such as LTE Cat-M1 [1]; others, like Narrowband IoT (NB-IoT) [1], are referred to as clean-slate technologies and do not retain full compatibility with existing technologies. As a result, these standards interfere with neighboring networks.

NB-IoT has emerged from a 3GPP study on cellular IoT technologies [4] and was first standardized as part of LTE Release 13 (Rel-13) [5]. It is designed to integrate with existing GSM and LTE networks and provide a long-range, energy-efficient mobile service, with a Maximum Coupling Loss (MCL) of 164 dB and up to 10 years of battery life. Additionally, NB-IoT supports massive device densities and guarantees delivery of high-priority exception reports within 10 seconds. In the future, NB-IoT will be part of the 5G architecture, providing support for mMTC use cases [6].

Whenever new mobile network standards are introduced, their performance is approximated in system-level simulations, which include the air interface, as well as higher protocol layers. In the case of NB-IoT, extensive simulations have already been performed for different scenarios [1,6].

NB-IoT Rel-13 has been implemented by MNOs and gained considerable interest; however, little attention has been paid to systematic real-life QoS evaluations. While some measurements have been done, not all of them were performed in a real-world environment, which is essential to verify the end-user QoS. Mozny et al. [7] analyzed attachment delay, latency, and message overhead under laboratory conditions. The analysis was based on five samples per measurement, which is a relatively low sample size. Khan et al. [8] performed long-term analysis of NB-IoT Signal-to-Noise Ratio (SNR) and Received Signal Strength Indicator (RSSI) on different floors of a university building. The evaluation showed that SNR and RSSI levels changed during the day. The authors proposed that this may be caused by LTE activity generated by mobile network users. Malik et al. [9] analyzed NB-IoT coverage in indoor, outdoor, and underground scenarios. The results indicated that NB-IoT worked well for indoor and outdoor scenarios, while underground coverage was limited to locations close to the base station, which is referred to as eNodeB in LTE systems. Martinez et al. [10] performed analysis on energy consumption, reliability, and latency in NB-IoT networks. While these measurements were performed in an operative network, the authors focused on the power consumption of NB-IoT, and they found no correlation between the repetitions caused by the Enhanced Coverage Level (ECL) and latency. A thorough search of the relevant literature revealed that only Basu et al. [11] performed detailed application layer QoS measurements in a commercial NB-IoT network. The results showed that decreasing signal levels resulted in increased latency and a reduction in throughput, but overall indicated a reliable performance of NB-IoT. However, the work considered a single tone downlink scenario, which did not align with the NB-IoT specification. Furthermore, only limited analysis has been performed on the physical NB-IoT network parameters and their influence on the application layer QoS.

This work expands on the previous publications by systematically examining both physical and application layer NB-IoT QoS parameters in a commercial mobile network. The influence of the physical QoS parameters and the underlying mechanisms on the end-user QoS is analyzed and compared against theory, as well as simulations. A long-term measurement allows concluding on how the radio conditions change between day and night. Moreover, this paper presents the suitability of NB-IoT in the following four use cases of smart metering applications in the uplink and downlink direction:

- Uplink transfer of individual sensor data from a single sensor
- Downlink transfer of individual commands
- Uplink transfer of small packets from many sensors
- Transfer of software updates in the downlink and bulk data to the cloud in the uplink

To the best of the authors’ knowledge, this paper contains one of the first systematic analyses of the relevant physical and application layer QoS parameters and contributing factors in a real NB-IoT network and is the first study of NB-IoT QoS in Germany.

The remainder of the paper is organized as follows: Section 2 describes the theoretical fundamentals of the NB-IoT architecture, as well as important QoS parameters. Section 3 explains the experimental setup and the measurement methodology. Section 4 presents the results of the QoS analysis. Section 5 discusses the suitability of NB-IoT in four different smart metering use cases. Finally, a conclusion is drawn in Section 6.

## 2. Narrowband IoT Fundamentals

Narrowband IoT is a clean-slate LPWAN standard specified by the 3GPP standardization organization. It was introduced in LTE Release 13 [5], with significant improvements made in Releases 14 and 15 [12]. NB-IoT is designated as a 5G mMTC enabler in Release 15 [6] and will thus stay relevant in future mobile networks. In this section, only the fundamental aspects of the NB-IoT Rel-13 standard are introduced, which are of relevance for the analysis in this paper. Additional details can be found in [1,13].

### 2.1. Deployment Modes and Resource Grid

**Figure 1:**
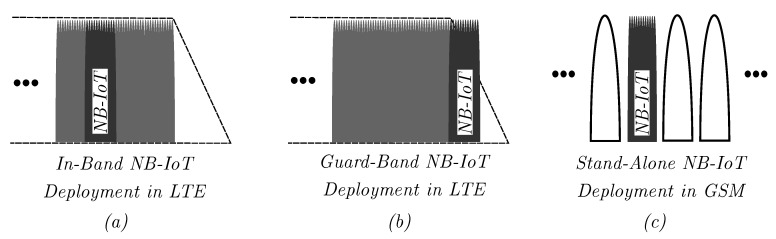
NB-IoT allows flexible deployment along existing mobile networks: (**a**) LTE in-band deployment, (**b**) LTE guard-band deployment, (**c**) GSM stand-alone deployment (adapted from [14]).

NB-IoT occupies a total system bandwidth of 180 kHz and can be deployed in-band within a single LTE Physical Resource Block (PRB) or operate in the LTE guard band on each side of the LTE carrier. Alternatively, it can occupy a single GSM carrier of 200 kHz in stand-alone mode, as shown in Figure 1.

**Figure 2:**
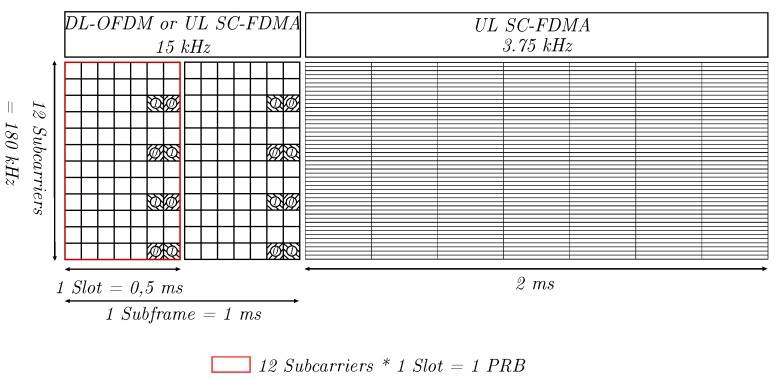
LTE resource grid. The NB-IoT Downlink (DL) is based on 15 kHz carrier spacing and OFDM, carrying reference symbols. The Uplink (UL) is based on 15 kHz or 3.75 kHz carrier spacing and SC-FDMA.

The integration of NB-IoT into existing 4G networks is facilitated by reusing the LTE resource grid as shown in Figure 2. The NB-IoT downlink is based on OFDM with 15 kHz carrier spacing and always uses all 12 subcarriers. Downlink resources are allocated as subframes, which span 1 ms and contain two slots with seven OFDM symbols each. As such, each allocated subframe contains two PRBs. The downlink radio channel is estimated using Narrowband Reference Symbols (NRS) that are present in the last two OFDM symbols of each slot. In the uplink direction, SC-FDMA is used, and the resource grid can be configured to use either 15 kHz or 3.75 kHz carrier spacing.

**Figure 3:**
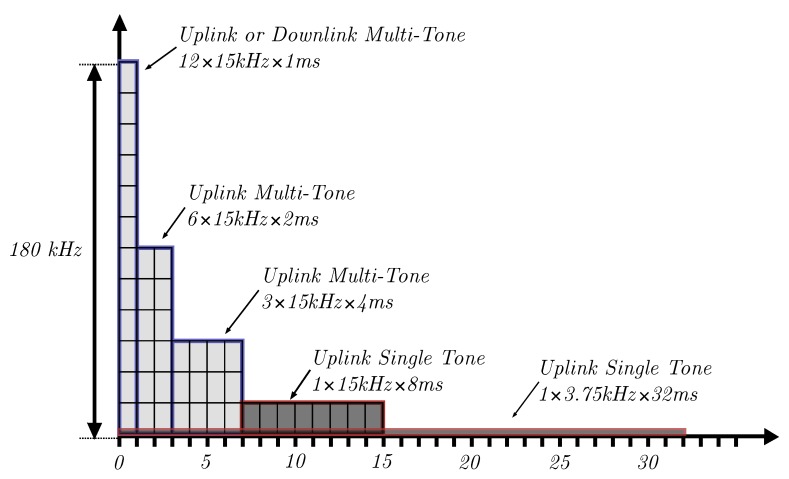
Resource allocation in NB-IoT uplink and downlink directions. Multi-tone allocations are highlighted in light gray with blue borders, and single tone allocations are dark gray with red borders.

Additionally, NB-IoT introduces the concept of Resource Units (RU) in the uplink, which allows allocating one or more subcarriers of a single PRB to different User Equipment (UE). These subcarriers are referred to as tones, and the resulting configurations are called single and multi-tone operation. The possible allocation schemes [15] are illustrated in Figure 3.

### 2.2. NB-IoT Physical Layer Radio Measurements

The LTE specification defines a set of radio QoS parameters, which are used to estimate the channel between the eNodeB and the UE [16]. This section introduces the parameters that are most relevant to evaluate end-user QoS, as well as their relationship to each other.

- The Reference Signal Received Power (RSRP) is a linear average of the power in Watts of the resource elements carrying NRS in a given frequency bandwidth. Since NB-IoT downlink is based on a 15 kHz carrier spacing, the RSRP is the power of a single 15 kHz NRS.
- The Received Signal Strength Indicator (RSSI) is a linear average of the total power in Watts received in the measurement bandwidth from all sources, including external interference, noise, and others. In NB-IoT, the measurement bandwidth is exactly one PRB or 180 kHz. The RSSI depends on the cell load; it increases with the number of allocated subcarriers.
- The Reference Signal Received Quality (RSRQ) describes the ratio between RSRP and RSSI, where both measurements shall be made over the same set of resource blocks:
  (1) $$RSRQ=\frac{RSSI[W]}{RSRP[W]}$$
- The Signal-to-Interference and Noise Ratio (SINR) is the ratio between the received signal level and the interference power ($P_{I}$) from external sources, as well as the effective noise power ($P_{N,eff}$). If all 12 Resource Elements (REs) of the broadband channel are occupied with the same signal power as the NRS, the narrowband and broadband SINR are identical.
  (2) $$SINR=\frac{RSRP[W]}{P_{I,15kHz}+P_{N,eff,15kHz}}=\frac{RSSI[W]}{P_{I,180kHz}+P_{N,eff,180kHz}}$$

The SINR and RSRQ parameters are related to each other via the subcarrier activity factor *x*, which defines the ratio of occupied REs in a Resource Block (RB): x=RE/RB. In an unloaded NB-IoT cell, only the NRS are active, so RE=2 and x=2/12, while a fully loaded cell uses all subcarriers (x=1).

The relation between the parameters SINR, RSRQ, and the number of occupied REs or the subcarrier activity factor was derived in [17]:

(3) $$SINR=\frac{12}{\frac{1}{RSRQ}-RE}=\frac{12}{\frac{1}{RSRQ}-12*x}$$

This relationship is validated in Section 4.4 by analyzing the RSRQ and SINR measurements of the NB-IoT UE, which allows concluding on the subcarrier activity factor *x*.

### 2.3. Data Rate and Latency Boundaries

NB-IoT dynamically adjusts to the radio conditions by configuring the Modulation and Coding Scheme (MCS), which is a combination of a modulation type and a coding rate applied to a given PRB. NB-IoT supports MCS 0 to 12, using QPSK or BPSK modulation [15] and a variable Transport Block Size (TBS). Higher MCS implies reduced coding redundancy and thus provides increased TBS at the same number of RUs. Additionally, the coverage range can be improved by applying signal repetitions $N_{Rep}$, which increase the receiver sensitivity. User data are transmitted via two physical channels, which are called the Narrowband Physical Uplink Shared Channel in Format F1 (NPUSCH F1) and the Narrowband Physical Downlink Shared Channel (NPDSCH).

When calculating an upper bound for the data rate in the uplink (NPUSCH F1) and downlink (NPDSCH) direction, the maximum possible MCS and TBS must be considered, which correlate to the minimum number of resource units ($N_{RU}$) and subframes ($N_{SF}$) used for the transmission [1]:

(4) $$NPUSCH\;multi\;tone:\;TBS_{max}=1000\;b,MCS_{max}=12\to N_{RU,min}=4$$

(5) $$NPUSCH\;single\;tone:\;TBS_{max}=1000\;b,MCS_{max}=10\to N_{RU,min}=6$$

(6) $$NPDSCH:\;TBS_{max}=680\;b,MCS_{max}=12\to N_{SF,min}=3$$

As such, the minimum time needed to transmit the maximum TBS without repetitions ($N_{Rep}$ = 1) can be calculated. In the uplink, the time needed to transmit one RU ($T_{RU}$) is 1 ms for a 180 kHz multi-tone transmission and 8 ms in the case of a 15 kHz single tone transmission. In the downlink direction, the time needed to transmit one subframe ($T_{SF}$) is 1 ms.

The minimum time needed to transmit 1000 b in uplink (NPUSCH F1) is:

(7) $$T_{min,15\mathrm{kHz}}=N_{rep}*N_{RU,min}*T_{RU}=1*6*8\;\mathrm{ms}=48\;\mathrm{ms}\;\mathrm{for}\;15\;\mathrm{kHz}\;\text{single tone}$$

(8) $$T_{min,180\mathrm{kHz}}=N_{rep}*N_{RU,min}*T_{RU}=1*4*1\;\mathrm{ms}=4\;\mathrm{ms}\;\mathrm{for}\;180\;\mathrm{kHz}\;\text{multi-tone}$$

The minimum time needed to transmit 680 b in downlink (NPDSCH) is:

(9) $$T_{min,180\mathrm{kHz}}=N_{rep}*N_{SF,min}*T_{SF}=1*3*1\;\mathrm{ms}=3\;\mathrm{ms}\;\mathrm{for}\;a\;180\;\mathrm{kHz}\;\mathrm{PRB}$$

**Figure 4:**
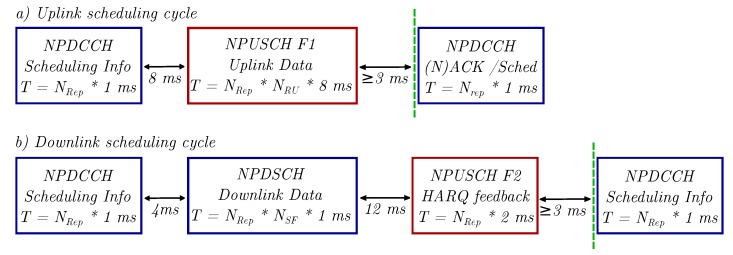
NB-IoT scheduling cycle for a single (**a**) uplink and (**b**) downlink transmission. Image adapted from [6].

NB-IoT employs a scheduling cycle to allocate resources to the individual UE, which reduces the physical layer effective data rates and results in the Medium Access Control (MAC) layer data rates. This scheme is illustrated in Figure 4a for an uplink and in Figure 4b for a downlink transmission. Periodic scheduling information is transmitted on the Narrowband Physical Downlink Control Channel (NPDCCH), and user data are sent via NPUSCH F1 and NPDSCH. NB-IoT uses Hybrid Automatic Repeat Request (HARQ) feedback for forward error correction and automatic retransmissions, which is transferred in the uplink on the NPUSCH in Format F2. The green dashed line marks the earliest possible start of a new scheduling cycle and defines the minimum time needed for the transmission of the TBS.

Accordingly, the upper boundaries for the MAC layer data rates can be calculated. These values are used as a benchmark for the throughput evaluations in Section 4.6 of the paper.

The maximum physical data rate ($R_{max}$) in NPUSCH is calculated as follows:

(10) $$T_{min,15\mathrm{kHz}}=60\;\mathrm{ms},TBS=1000\;b\to R_{max}=TBS/T_{min}=16.7\;\mathrm{kbps}\;\mathrm{for}\;15\;\mathrm{kHz}\;\text{single tone}$$

(11) $$T_{min,180\mathrm{kHz}}=16\;\mathrm{ms},TBS=1000\;b\to R_{max}=TBS/T_{min}=62.5\;\mathrm{kbps}\;\mathrm{for}\;180\;\mathrm{kHz}\;\text{multi-tone}$$

On the other hand, $R_{max}$ in NPDSCH is:

(12) $$T_{min}=25\;\mathrm{ms},TBS=680\;b\to R_{max}=TBS/T_{min}=27.2\;\mathrm{kbps}\;\mathrm{for}\;a\;180\;\mathrm{kHz}\;\mathrm{PRB}$$

In NB-IoT, there are multiple factors that contribute to the total system latency. Liberg et al. [1] performed system-level simulations to evaluate the latency under different signal conditions and deployment modes, as shown in Table 1. The simulation found that at 144 dB coupling loss, NB-IoT provided a low latency of about 300 ms, which was mostly determined by the time needed to access the network and acquire the necessary configuration information. At the MCL of 164 dB, the latency was dominated by the signal repetitions used for coverage extension, but still met the limit of 10 s for delivering exception reports, as defined by 3GPP [4]. The differences between the individual deployment modes could be attributed to the output power restrictions that apply for in-band and guard-band installations. Overall, the latency estimation can serve as a useful benchmark for real-live evaluations.

### 2.4. Receiver Sensitivity, ECL, MCL, and Transmission Power Control in NB-IoT

The receiver sensitivity ($P_{RX,min}$) defines the minimum input power level at the receiver antenna port related to a QoS threshold. This threshold is commonly specified at a Block Error Rate (BLER) of 10% for the NPUSCH and NPDSCH NB-IoT channels [1]. The BLER depends on the MCS and the amount of symbol repetitions ($N_{Rep}$) applied for a given Signal-to-Noise Power Ratio (SNR). Accordingly, $SNR_{min}$ defines the minimum power ratio needed in order to achieve this QoS target. In the first step, the thermal noise needs to be considered:

(13) $$P_{N}=10*log\left(kTB/1\mathrm{mW}\right)$$

where *k* is the Boltzmann constant, *T* is the temperature, and *B* is the bandwidth.

The effective noise floor ($P_{N,eff}$) takes into account the Noise Figure (NF) of the receiver front-end, which adds noise to the thermal noise power ($P_{N}$):

(14) $$P_{N,eff}=P_{N}+NF$$

The analysis of the measurement results requires the calculation of the effective noise floor of the RSRP, the RSSI, and the 15 kHz single-tone NPUSCH. These can be calculated at room temperature, considering the corresponding Bandwidths (B) and the assumed NFs of UE and eNodeB:

(15) $$P_{N,eff,NPUSCH}=P_{N,15\mathrm{kHz}}+NF_{eNodeB}=-132.2\;\mathrm{dBm}+5\;\mathrm{dB}=-127.2\;\mathrm{dBm}$$

(16) $$P_{N,eff,RSSI,DL}=P_{N,180\mathrm{kHz}}+NF_{UE}=-121.4\;\mathrm{dBm}+7\;\mathrm{dB}=-114.4\;\mathrm{dBm}$$

(17) $$P_{N,eff,RSRP,DL}=P_{N,15\mathrm{kHz}}+NF_{UE}=-132.2\;\mathrm{dBm}+7\;\mathrm{dB}=-125.2\;\mathrm{dBm}$$

Using the previous definitions, the receiver sensitivity ($P_{RX,min}$) is defined as:

(18) $$P_{RX,min}=P_{N}+NF+SNR_{min}=P_{N,eff}+SNR_{min}$$

In NB-IoT, the receiver sensitivity is improved by applying signal repetitions [15]. Theoretically, each doubling of the repetitions increases the sensitivity by 3 dB due to coherent addition of the symbols and incoherent addition of thermal noise. NB-IoT employs up to 128 repetitions in the uplink and 2048 repetitions in downlink direction. The large number of repetitions in downlink compensates the higher effective downlink noise floor in order to balance the link budget. However, accumulating the signal repetitions takes time and is thus a tradeoff between latency and sensitivity.

The appropriate number of repetitions is adjusted dynamically by the eNodeB, which assigns an ECL to each device based on the received uplink and reported downlink signal level, where a higher ECL corresponds to more problematic radio conditions and a higher number of repetitions. The exact number of repetitions per ECL is defined by the MNO. This paper refers to the three available ECL levels as ECL0, ECL1, and ECL2.

The MCL is a common metric to evaluate the coverage of a radio access technology and is calculated as the difference between the transmitted power level at the antenna connector ($P_{TX}$) and the receiver sensitivity. Since the downlink NRS are used for channel estimation, their constant transmit power is indicated to the UE by the eNodeB and used for all physical channels. In the uplink direction, the transmission power depends on the coverage situation. For up to two repetitions, the transmission power is a function of multiple cell parameters including coupling loss. For more than two repetitions, the cell-specific maximum transmission power on slot *i* ($P_{CMAX,c(i)}$) is used. This paper refers to the transmit power of the UE as $P_{TX,UE}$ [18]. The MCL is thus calculated as:

(19) $$MCL=P_{TX}-P_{RX,min}$$

Table 2 shows a reference link budget configuration with the uplink $N_{Rep,UL}$ = 32 and the downlink $N_{Rep,DL}$ = 128 achieving the minimum requirement of MCL = 164 dB as defined by the International Mobile Telecommunications 2020 (IMT-2020) standard [19], which specifies the requirements for upcoming 5G systems.

## 3. Experimental Setup and Methods

The complexity of mobile networks makes a systematic QoS evaluation challenging for end-users. In most cases, the measurements performed by the base station cannot be obtained from the network. As a result, QoS parameters such as the received signal level and quality, SNR, eNodeB transmission power, as well as various radio configuration parameters like the number of ECL repetitions are not available to estimate the network performance. However, modems perform extensive lower layer measurements for channel estimation, which can be employed to understand the system behavior under different coverage situations. Furthermore, the application layer measurements can be used to analyze the data rates and latency. In this section, the setup used for the experiments is presented.

**Figure 5:**
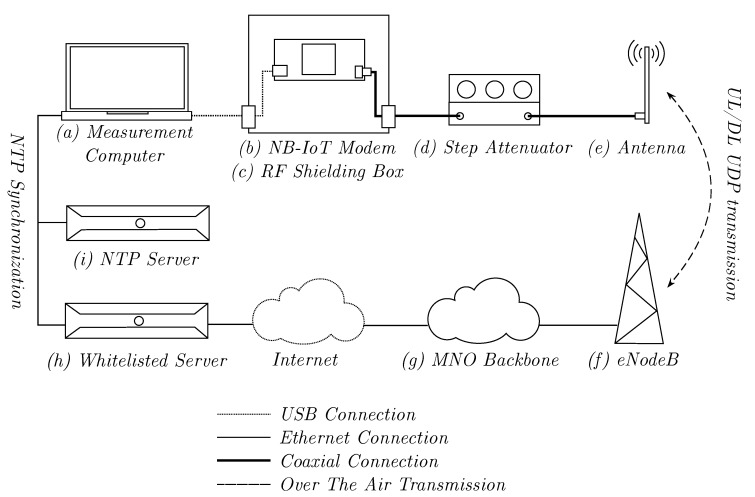
The measurement architecture used for the physical and application layer evaluations.

All measurements in this research were performed from an end-user perspective, without access to the base station or control of the serving network. As such, the experiments could be reproduced by application developers and could be employed to estimate the QoS that could be expected for a given use case in different coverage scenarios. Most physical radio parameters, such as ECL and the number of repetitions, were controlled by the serving network or the eNodeB. One notable exception was the UE transmission power ($P_{TX,UE}$), which was set by the UE algorithm, but was subject to cell-specific and user limits. In this evaluation, the maximum user limit of 23 dBm was used. Figure 5 shows the architecture used for measuring the physical and application layer NB-IoT QoS parameters.

The architecture consisted of the following elements:

(a) A measurement computer controlled the NB-IoT modem via a USB connection. A UDP client was running on the computer to send and receive test packets.
(b) The NB-IoT modem connected to the public NB-IoT network and performed lower layer measurements. The Exelonix NB|DEV kit [20], which is based on the uBlox SARA-N211 modem [21], was selected for the physical layer measurements due to its wide range of reported QoS values.
(c) A RF shielding box prevented the mobile carrier signal from coupling into the modem board when high attenuation was applied.
(d) A Wavetek 5080.1 step attenuator (0–81 dB attenuation, $f_{max}$ = 1000 MHz) allowed simulating different signal levels.
(e) A Delock 88571 omnidirectional antenna (A = −0.8–0.2 dBi) was connected via a coaxial cable to the step attenuator.
(f) The eNodeB was the MNO base station providing the NB-IoT carrier signal.
(g) The MNO backbone contained provider internal functionality, such as subscriber management and packet routing.
(h) A whitelisted server was required by the MNO as a packet destination for security reasons. The server ran a UDP server to send and receive test traffic.
(i) A Network Time Protocol (NTP) server was part of the same local network as the measurement computer and the whitelisted server. It was used to synchronize the real-time clocks and enabled high-precision latency measurements.

The measurements were performed in a public NB-IoT network in Germany, which was installed as a guard-band deployment in Band 20 (800 MHz). All measurements were conducted indoors with a Non-Line Of Sight (NLOS) connection to the eNodeB. The modem was kept stationary during the measurements. Since the modem implemented a full network stack, all IP connections were created between the whitelisted server and the modem itself. As such, the modem served as the source for all uplink packets, while the whitelisted server was the source for all downlink packets. Two groups of experiments were performed: physical and application layer QoS measurements.

(a) In the first group of measurements, the physical layer QoS was analyzed using the downlink parameters provided by the NB-IoT modem. For each 5 dB attenuation step, 30 UDP packets were transmitted via the user plane both in the uplink and downlink direction. The packets were identical in the uplink and downlink and included UDP, IP, PDCP, RLC, and MAC headers. Each packet carried a 27 byte ASCII payload that was comprised of the following elements:
  - An indicator for uplink or downlink transmissions
  - A sequence number, which was incremented by one for each packet
  - The current attenuation step in dB
  - A timestamp in milliseconds
  For each transmission, all available QoS parameters were collected along with the timestamps of both endpoints. Between each measurement point, a delay was introduced to prevent two measurements from interfering with each other.
(b) The second group of measurements was conducted to examine the application layer QoS. There were two transmission categories that covered most IoT use cases:
  - Uplink and downlink transfer of small, individual packets (e.g., sensor data) with a focus on latency
  - Uplink and downlink transfer of a continuous data stream (e.g., a software update) with a focus on throughput
  The first measurement employed the previously acquired packet timestamps to analyze the total system latency for individual packets at varying signal levels, which were simulated using the step attenuator. This allowed identifying conditions that significantly affected the QoS.
  In the second measurement, the maximum data rate of NB-IoT was evaluated in different coverage situations, which were also enforced using the attenuator. The measurement employed UDP, which is a common protocol in IoT applications and lacks mechanisms that could influence the measurement like congestion and flow control. The following parameters were adjusted to evaluate a wide range of potential use cases:
  - Signal level (10 dB steps)
  - Packet size (8 B, 64 B, 512 B, 1024 B)
  - Uplink and downlink direction
  For each combination of attenuation step, packet size, and direction, 100 UDP packets were sent to simulate a continuous data transmission. Since the Exelonix NB|DEV module imposed a packet size limit of 237 bytes and restricted the packet rate, the measurement was conducted using the PyCom GPy 4 NB-IoT modem [22]. The NB-IoT standard offers optimizations for non-IP communication, which reduce the protocol overhead by omitting IP and UDP headers. At the time of writing, these optimizations were not publicly available, so only IP-based transmissions were evaluated.

## 4. Results

In this section, the measurement results are presented and analyzed in terms of physical and application layer QoS. For each evaluation, the measurement procedure is described. Afterwards, the observations are discussed, and an initial conclusion is drawn.

### 4.1. PHY Measurements: Coupling Loss

The first measurement explored the RSRP and RSSI signal level parameters. This evaluation was essential to find a suitable representation of path loss for later measurements. A wide range of signal levels was simulated by manually introducing attenuation in 5 dB steps until the signal was lost. For each attenuation level, 30 packets were sent, and the downlink RSRP and RSSI (DL-RSRP and DL-RSSI) were recorded for each packet.

**Figure 6:**
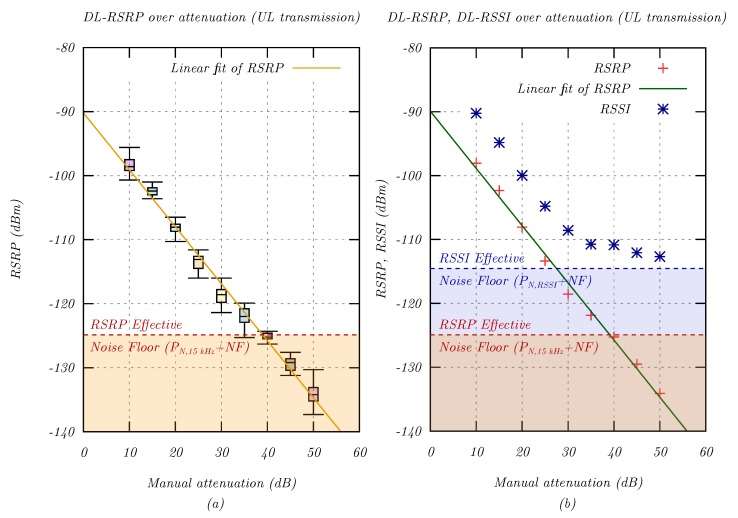
Analysis of different signal level measurements as an expression for coupling loss. (**a**) Distribution of RSRP signal levels for artificial attenuation in 5 dB steps. (**b**) Comparison between RSRP and RSSI for various levels of artificial attenuation.

Figure 6a shows that the RSRP was proportional to the coupling loss under all conditions, including signal levels below the effective noise floor (see Section 2.4). The ability to measure signal levels below the noise floor was enabled by coherently adding signal repetitions, which increased the receiver sensitivity. This measurement confirmed the sensitivity of the SARA-N211 modem of −135 dBm [23].

Figure 6b compares the RSRP and RSSI for different signal levels. While the RSRP was proportional to coupling loss, the RSSI converged to the 180kHz effective noise floor $P_{N,eff,180kHz}$ = −114.4 dBm. Since the RSSI included signal, noise, and interference power, it could not fall below the effective noise floor. As such, it was unsuitable for coupling loss evaluations.

Table 3 provides additional statistical detail on the RSRP and RSSI measurements. The RSRP mean values ($\mu_{RSRP}$) followed the manual attenuation closely for all signal levels, with a maximum error of 3% at 50 dB attenuation. On the other hand, the RSSI mean values ($\mu_{RSSI}$) converged to the effective noise floor, with a minimum average value of −112.2 dBm at 50 dB attenuation. As expected, the standard deviation of the 15 kHz RSRP signal level ($\sigma_{RSRP}$) was higher than for the RSSI ($\sigma_{RSSI}$), which had a larger bandwidth of 180 kHz.

In the 3GPP specification, the coupling loss was estimated using the RSRP measurement [18]:

(20) $$Coupling\;Loss\;CL\left[dB\right]=P_{TX,NRS}-RSRP_{filtered}$$

where $RSRP_{filtered}$ is the higher layer filtered RSRP.

The power $P_{TX,NRS}$ allocated to an NRS depends on the system configuration [18] and was approximated hereafter proportional to the bandwidth:

(21) $$P_{TX,NRS}\approx P_{TX,NRS}+10log\left(1/12\right)=35\;\mathrm{dBm}-10.8\;\mathrm{dB}=24.2\;\mathrm{dBm}$$

The minimum observed DL-RSRP value during the measurement was −137.3 dBm at 50 dB attenuation. The maximum observed MCL could therefore be approximated:

(22) $$MCL_{max,observed}\approx 24.2\;\mathrm{dBm}-\left(-137.3\;\mathrm{dBm}\right)=161.5\;\mathrm{dB}$$

In a following evaluation related to the exception report latency, a minimum DL-RSRP level of −139.3 dBm was measured, which resulted in a $MCL_{max,observed}$ of 163.5 dBm. As such, the MCL closely matched the 3GPP specification [4].

Overall, the RSRP and RSSI measurements behaved as expected. There was a slight reduction from the 3GPP specified MCL of 164 dB, which could be attributed to external interference. The MNO could compensate this by configuring more ECL repetitions, which in turn would increase the latency.

### 4.2. PHY Measurements: Coverage Extension

In this evaluation, the selection of different extended coverage levels (ECLs) by the NB-IoT network was examined. This allowed understanding how NB-IoT used signal repetitions to adjust to changing signal levels. Various coverage situations were simulated using the attenuator. For each 5 dB attenuation step, 30 packets were transmitted in the uplink and downlink direction, and the selected ECL was recorded for each packet.

**Figure 7:**
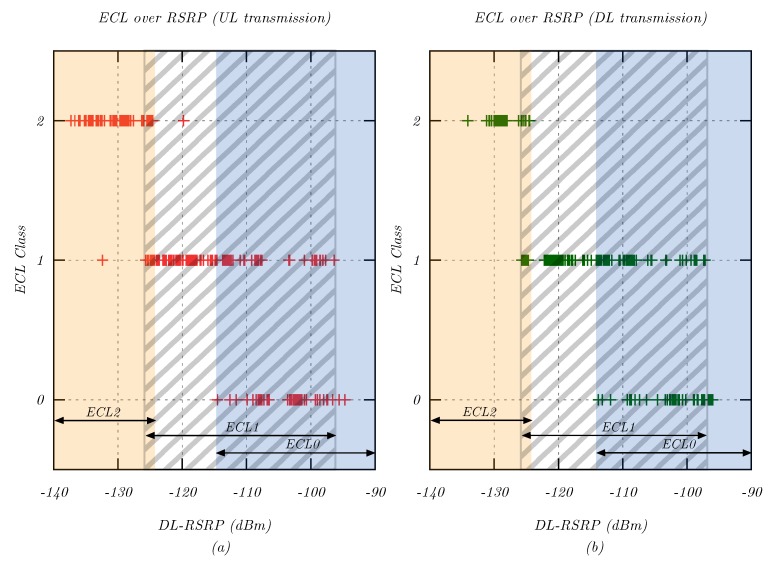
Relationship between ECL selected by the modem and downlink RSRP signal level during (**a**) uplink and (**b**) downlink transmissions.

Figure 7 illustrates the ECL assigned to the UE by the eNodeB depending on the DL-RSRP signal level. In general, higher ECL were selected for lower RSRP levels. There was an overlap between the individual ECL regions, which indicated a hysteresis in the ECL selection algorithm of the MNO. This technique avoided constant switching between different ECL levels for minor variations in the signal level. The network used ECL 0 for RSRP signal levels above approximately −115 dBm, ECL1 between −126 dBm and −97 dBm, and ECL2 below −125 dBm RSRP. This allocation indicated that ECL2 was selected for detecting signals at or below the effective noise floor $P_{N,eff}=P_{N}+NF$.

### 4.3. PHY Measurements: Transmission Power

This measurement analyzed the transmission power $P_{TX,UE}$ selected by the modem in different coverage situations. Accordingly, conclusions could be drawn on the signal level needed for output power reduction. Various signal levels were simulated in 5 dB steps using the attenuator, and 30 packets were sent for each step in uplink and downlink direction. For each packet, $P_{TX,UE}$, as well as the RSRP signal level were recorded.

**Figure 8:**
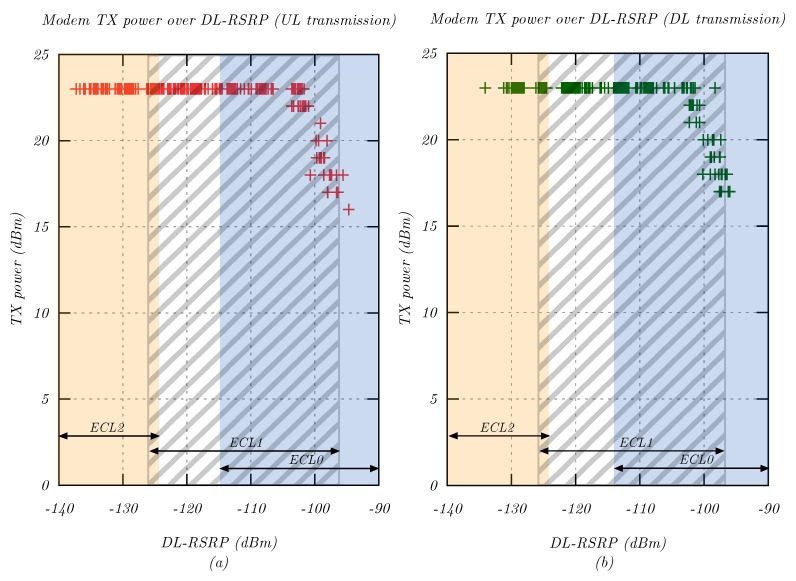
UE transmit power $P_{TX,UE}$ as a function of downlink RSRP signal level for (**a**) uplink and (**b**) downlink transmissions.

Figure 8 shows the transmission power $P_{TX,UE}$ of the NB-IoT UE at different signal levels. For RSRP levels below −102 dBm, the maximum cell-specific output power of $P_{CMAX,c}$ = 23 dBm was used. Above this level, the UE reduced its output power in 1 dB steps. This technique conserved power for battery operated devices, while maintaining a reasonable performance at high received signal levels. The power reduction depended on the estimated coupling loss, which was calculated using the current RSRP value [18]. Overall, this measurement indicated that an improvement in received signal level (e.g., by installing an external antenna) could significantly improve battery life.

### 4.4. PHY Measurements: Signal Quality Analysis

In this section, the signal quality parameters SNR, SINR, and RSRQ were used to analyze the Interference Margin (IM), the noise figure of the modem, as well as the cell load during the measurements. Various signal levels between excellent coverage and total signal loss were simulated in 5 dB steps using the attenuator. For each attenuation step, 30 packets were sent in the uplink and downlink, and the RSRP, SNR, and RSRQ were recorded. Additionally, a long-term measurement was conducted over 24 h. Every minute, one uplink and one downlink message were sent for a total of 1440 measurements per direction. For each packet, the SNR, RSRP, RSSI, and latency measurements were recorded.

**Figure 9:**
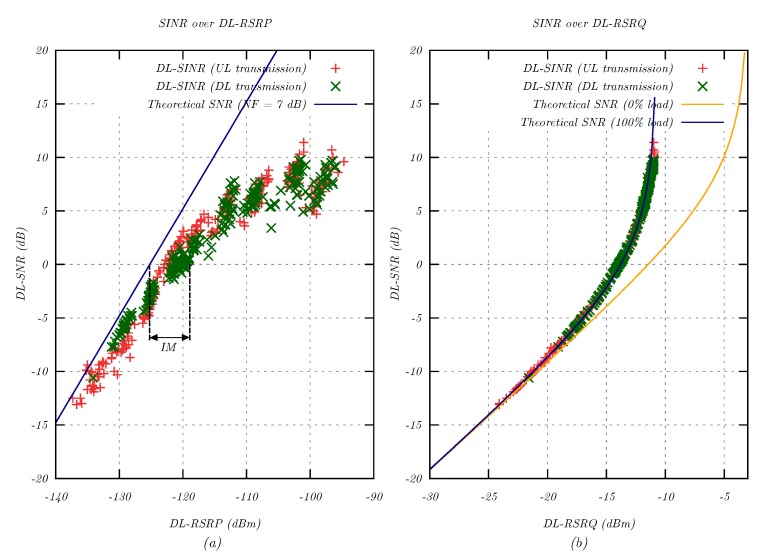
Analysis of the DL-SINR due to the presence of network interference for varying attenuation. (**a**) Measured DL-SINR over reported RSRP. (**b**) Measured DL-SINR over RSRQ. The solid lines show the theoretical curves for x = 2/12 (0% load) and x = 1 (100% load) according to Equation (3).

Figure 9a compares the theoretical SNR to the measured SINR as a function of RSRP. The SNR could be derived for an effective noise floor $P_{N,eff,15kHz}$ = −125.2 dBm and a noise figure NF = 7 dB:

(23) $$SNR\left[dB\right]=RSRP-P_{N,eff,15kHz}=RSRP-P_{N,15\mathrm{kHz}}-NF=RSRP-125.2\;\mathrm{dBm}$$

The SINR additionally included interference, so the SNR was an upper bound for the measured SINR:

(24) $$SINR\left[dB\right]=SNR\left[dB\right]-10log\left(1+\frac{P_{I,15kHz}\left[W\right]}{P_{N,eff,15kHz}\left[W\right]}\right)$$

The IM, which was considered in the link budget in Section 2.4, could then be derived as:

(25) $$IM=10log\left(1+\frac{P_{I,15kHz}\left[W\right]}{P_{N,eff,15kHz}\left[W\right]}\right)$$

Figure 9a shows an offset between the theoretical SNR and the measured SINR samples, which was an indication of the presence of network interference during the measurement. The resulting IM of 2–6 dB as defined in Equation (25) increased the effective noise floor and reduced the MCL in a real NB-IoT network. Furthermore, Figure 9a provides an upper bound of 7 dB for the NF, since a higher NF would shift the slope of the theoretical SNR to the right and violate the condition that SNR was an upper bound for SINR.

In Figure 9b, the theoretical SNR for 0% cell load (*x* = 2/12) and 100% cell load (*x* = 1) is compared to the measured SINR values for varying RSRP signal levels. The measurement matched the theoretical curve for 100% load (see Equation (3)), which confirmed that all 12 subcarriers were allocated in every modem QoS measurement. Since all modem measurements were performed in the downlink, this verified the 3GPP NB-IoT specification [15] that required downlink resources to be allocated in subframes over the full system bandwidth.

**Figure 10:**
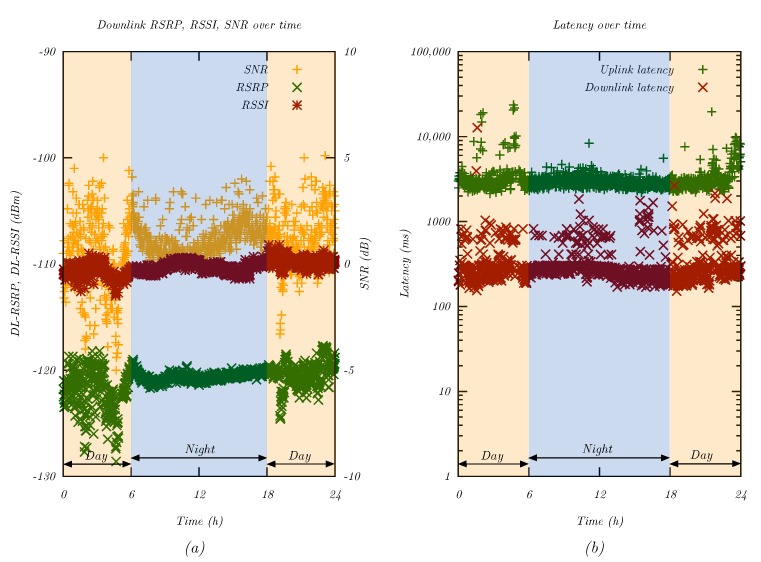
Long-term measurement of NB-IoT QoS parameters over a full day. (**a**) Signal quality parameters over one day. (**b**) Uplink and downlink latency over 24 h.

On the other hand, Figure 10a illustrates the signal conditions over a period of 24 h. The measurement points were filtered to only include one cell tower. There were significant fluctuations in the RSRP level, which reflected changes in the signal power received by the modem. These changes could be the result of human activity and created an effect similar to small-scale fading. At night, the signal conditions stabilized dramatically. As such, UE in extreme coverage might benefit from collecting the sensor values during the day and transmitting at night.

Figure 10b compares uplink and downlink latencies over time. Once again, the measurement points were filtered to only include one cell tower. Despite the significant changes in the RSRP and SNR levels shown in Figure 10a, the latency remained relatively constant. While there were latency spikes in the downlink, these values were well within the 10 s latency requirement of 3GPP [4].

### 4.5. Application Layer Performance: Latency

In the first application layer measurement, the factors impacting system latency were analyzed under varying coverage situations. The total system latency is the most important QoS criterion for small and infrequently transmitted packets, such as sensor data. Similar to previous evaluations, the signal level was varied in 5 dB steps using the attenuator, and 30 packets were sent per attenuation step and per direction. In a first analysis, the effect of the ECL mechanism on the uplink and downlink latency was considered. For this purpose, packets were grouped by their ECL class and analyzed in terms of latency.

**Figure 11:**
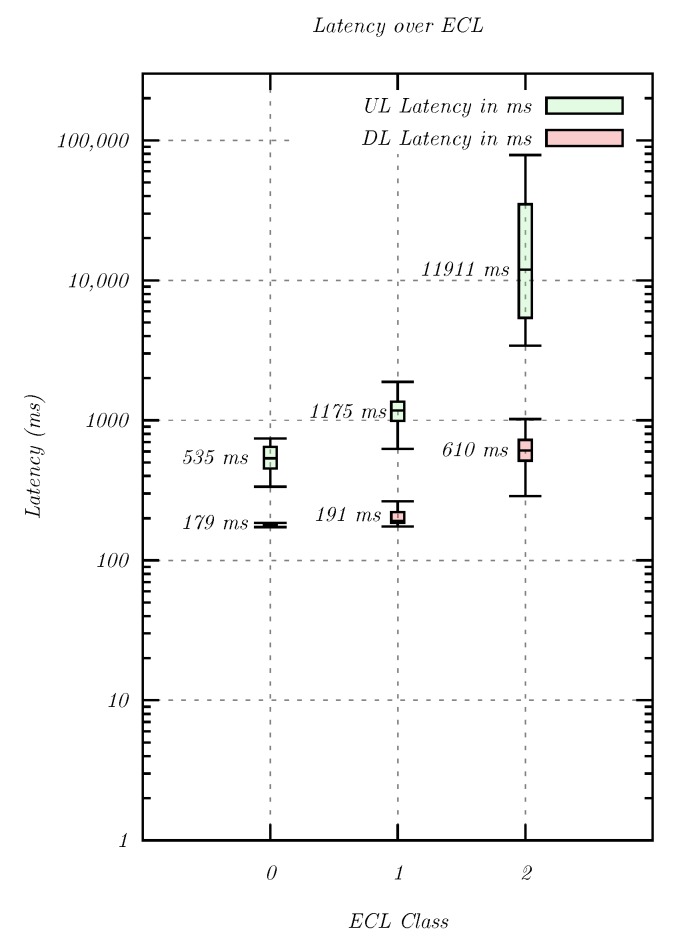
The influence of ECL classes on the total system latency. The median latency value is given for each ECL and transmission direction.

Figure 11 illustrates the effect of different ECL levels on the total system latency. The measurement confirmed that higher ECLs, and thus more signal repetitions, had an impact on the total system latency. The latency rise between ECL0 and ECL1 was less significant than between ECL1 and ECL2, which indicated a more drastic increase in the repetition number in the latter case. Furthermore, there was a significant difference between the latency in uplink and downlink direction. This could be explained by the allocation of 1 ms subframes over 12 subcarriers in the downlink and 8 ms RUs on a single tone in the uplink, which overcompensated the higher number of repetitions in the downlink direction. In order to obtain a better understanding of the latency contribution of the individual systems, the round-trip-time between the whitelisted server and the MNO backbone (see Figure 5h,g) was measured to be 17.776 ms (N = 30, σ = 0.163 ms), which indicated a latency of about 9 ms. As such, the latency contribution of the Internet link remained below 5% of the total system latency for all use cases.

The second analysis was concerned with the influence of the RSRP signal level on the total system latency. In addition to transferring regular user data, NB-IoT implements the ability to send exception reports, which are high-priority messages that can be used for reporting an alert condition. The 3GPP specified a maximum latency of 10 s for waking up the modem and delivering an exception report [1,4]. In order to evaluate if exception reports could reduce the system latency, the modem was configured to send 30 exception reports per attenuation step in the uplink direction. For each step, packets were grouped together and assigned to their average signal level, and the latency was analyzed.

**Figure 12:**
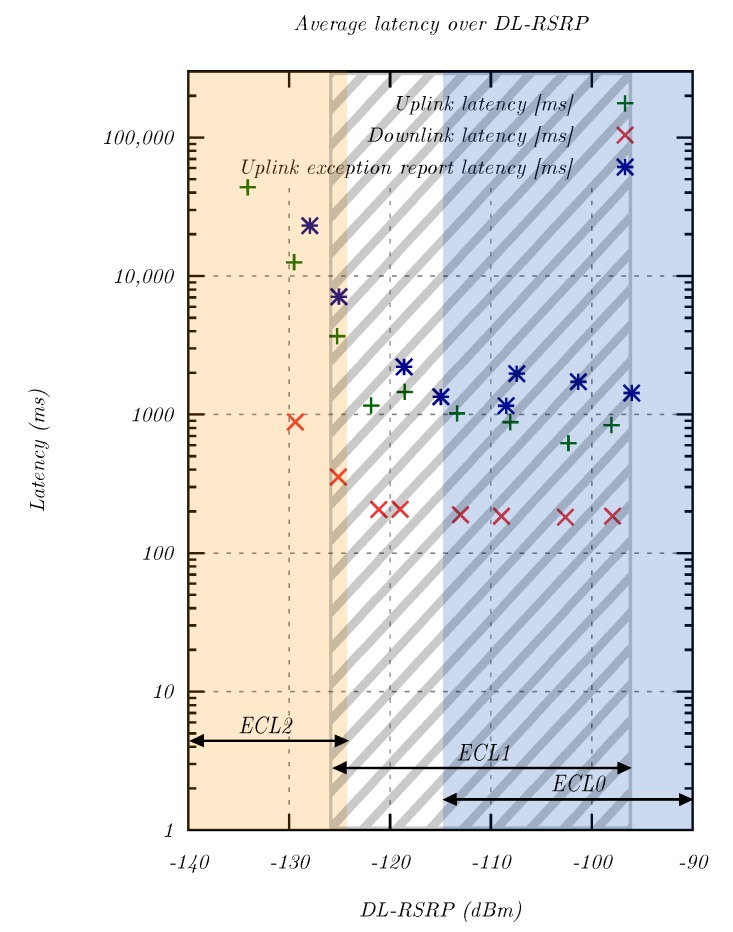
The total system latency as a function of RSRP signal level in the uplink and downlink direction for regular user data and exception reports.

Figure 12 confirms the relationship between the RSRP signal level and latency. In both the uplink and downlink direction, the latency rose significantly below the noise floor. There were two main reasons for this growth: (a) similar to LTE, NB-IoT used MCS to adapt to varying channel conditions; with lower MCS, the coding redundancy was increased, and the TBS decreased, which required more RU per data volume; (b) additionally, ECL2 was employed for signals below the noise floor as discussed in Section 4.2.

The comparison of the latency of regular user traffic and exception reports showed that there was no latency benefit, and the 3GPP target of 10 s was still exceeded for signals significantly below the noise floor. Further research is required to identify what was causing this behavior. A possible reason might be that exception reports had not yet been implemented in this NB-IoT network. Overall, the evaluation revealed a significant number of uplink samples with tens of seconds of latency in the uplink direction, which must be considered during application development.

### 4.6. Application Layer Performance: Data Rate

The second application layer evaluation was focused on the data rates provided by NB-IoT under different signal situations. This parameter was especially relevant for continuous data streams. The measurement considered the application layer goodput, which was the amount of user data that could be transmitted per second, as well as throughput, which also included protocol overhead of lower layers. Different signal levels were simulated in 10 dB steps using the attenuator, and 100 packets were transmitted for each attenuation step, in both uplink and downlink, as well as for different packet sizes of 8 B, 64 B, 512 B, and 1024 B to simulate multiple use cases.

**Figure 13:**
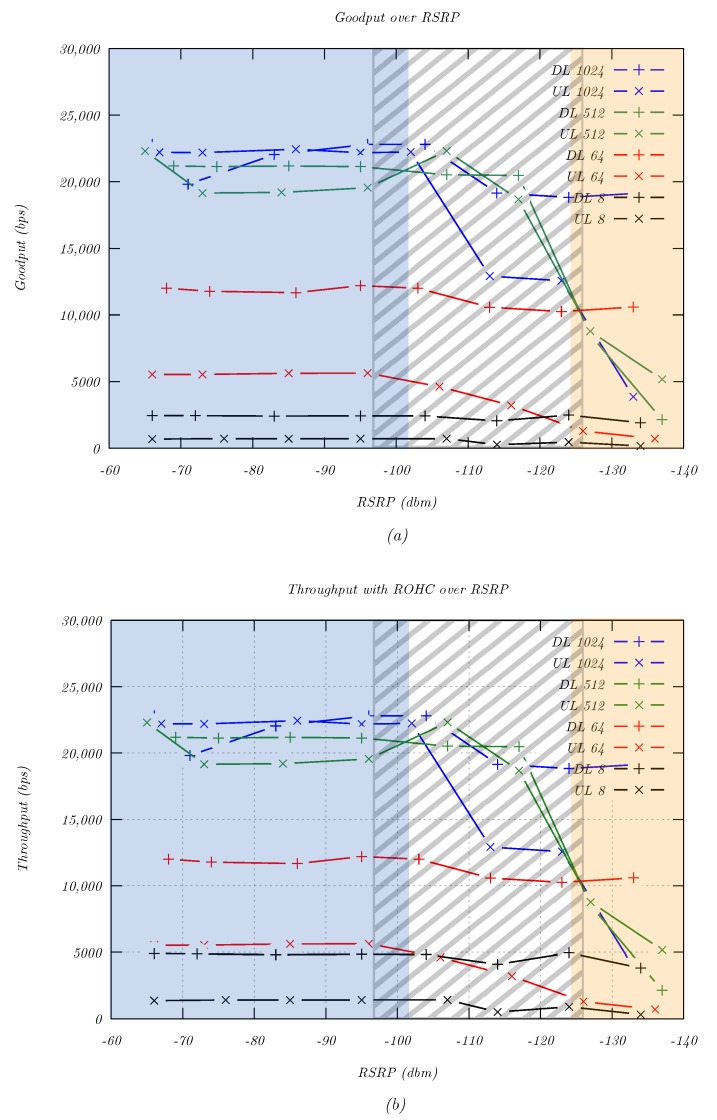
Maximum NB-IoT data rates for various packet sizes and signal levels. (**a**) Goodput excluding protocol headers for different packet sizes. (**b**) Throughput including protocol overhead for different packet sizes. The ECL regions are marked in blue (ECL0), striped (ECL1), and yellow (ECL2).

Figure 13a illustrates the goodput for different packet sizes and signal levels in the uplink and downlink direction. The highest data rates were possible with large packet sizes due to the comparatively small overhead. In uplink direction, 22.4 kbps could be achieved for 1024 byte packets, while the downlink provided up to 22.8 kbps. The data rates remained nearly constant until about −115 dBm RSRP, which allowed installation of NB-IoT UE in a wide variety of locations.

For smaller packets, the maximum goodput was significantly lower in both uplink and downlink direction. This was a result of the protocol overhead consuming larger portions of the total packet size. Furthermore, there was a large asymmetry between uplink and downlink data rates. This could be confirmed by analyzing the throughput, which consisted of the goodput plus the bandwidth consumed by the overhead:

(26) $$Throughput=Goodput*\left(1+\frac{Overhead}{Payload}\right)$$

For NB-IoT, the following lower layer protocol headers must be considered: MAC 2 bytes, RLC 2 bytes, and PDCP 5 bytes [1]. For IP and UDP, which would normally consume 20 bytes and eight bytes, respectively, NB-IoT PDCP implements Robust Header Compression (ROHC) [24]. During signaling, a compression context was set up between UE and eNodeB, which reduced the size of the IP and UDP header to a minimum of two bytes. As such, the total protocol overhead was decreased to 11 bytes. Using Equation (26), the throughput was calculated and is shown in Figure 13b.

The results showed a maximum throughput of 22.6 kbps in the uplink direction, which exceeded the limits of a single tone transmission. At the same time, this value was much lower than the multi-tone maximum of 62.5 kbps. In the downlink direction, the throughput of 23.0 kbps was well aligned with the theoretical limit of 27.2 kbps (see Section 2.3). The comparison with the goodput highlighted that for very small packets, the overhead was the most significant part of the physical data rate. For example, an 8 byte UDP packet experienced a total overhead of 11 bytes, or 137.5% of the payload. As such, transmissions of small packets (e.g., sensor data) could benefit from the non-IP optimizations of the NB-IoT standard, which further reduce the overhead.

## 5. Discussion

In this section, the real-life performance of NB-IoT is discussed and correlated with the 3GPP requirements [4] and existing system-level simulations [1]. Table 4 provides an overview of the most relevant QoS parameters, which were used to discuss the suitability of NB-IoT in different scenarios. For example, in smart metering applications, four transmission categories were identified depending on the functionality of the end device:

- Uplink transfer of individual sensor data from a single sensor
- Downlink transfer of individual commands
- Uplink transfer of small packets from many sensors
- Transfer of software updates in the downlink and bulk data to the cloud in the uplink

The first transmission category corresponded to a typical IoT use case, which was a single sensor transmitting data to the cloud for further processing and storage. Sensors are commonly deployed in hard to reach locations, such as basements and metal enclosures. NB-IoT was designed to provide long-range communication with 164 dB MCL. While this coverage was confirmed in the simulations as shown in Table 4, the measurements showed that the MCL could be reduced by interference, with an observed best case MCL of 163.5 dB.

The second important QoS criterion was latency. For NB-IoT, an exception report latency of 10 s was defined, which was fulfilled in a simulated environment at 8.0 s. In most real-world applications however, the latency of regular user traffic is of interest. NB-IoT provided a low user traffic latency at high signal levels with an uplink median of 535 ms for ECL0 transmissions. Below the noise floor, ECL2 was employed, and the latency increased significantly to a median of 11.2 s. Since there were many samples with a latency of tens of seconds, the application must be designed accordingly. For applications that employ exception reports, no latency benefit was found, which suggested that this functionality may not have been implemented in the network under investigation. Furthermore, the high repetition count (up to 128) and corresponding high output power (up to 23 dBm) decreased the battery life of sensors without a mains connection. Since the long-term measurement revealed more stable radio conditions during night time, it might be beneficial for sensors in extreme coverage to collect sensor readings and transmit them at night. No packet loss was observed for transmissions of individual packets, which relaxed the need for application layer retransmissions.

In the second category, an actuator received commands from a control server on the Internet. The traffic patterns and QoS requirements matched those of the sensors closely, except that commands were transferred in the downlink direction. As such, most of the QoS conclusions made for the transmission of sensor data also applied to actuators. For good signal levels with ECL0, NB-IoT provided very low median downlink latencies of 179 ms. In extreme coverage scenarios with ECL2, the target latency was still satisfied at a median of 610 ms. As such, NB-IoT fulfilled the need for transferring commands to actuators.

For the third category, a deployment of many sensors in a limited area was considered. One NB-IoT UE could aggregate and transmit all sensor readings. In this use case, many small packets would be sent within a short period of time, so the system bandwidth was an important QoS characteristic. For eight byte packets, the maximum uplink goodput was limited to 712 bps, while the corresponding throughput was at 1.7 kbps. This could be explained by the significant protocol overhead of 137.5%. As such, reducing the overhead and aggregating sensor data prior to transmission could significantly increase the efficiency. Overall, the sensor aggregation scenario was a prime use case for the NB-IoT non-IP optimizations, which were not yet supported by all networks.

The final category of sending software updates to end devices and bulk data to the cloud is challenging for many LPWAN technologies, since it requires the transmission of a large data volume in a certain time frame to prevent timeouts. As such, the maximum goodput rate is of interest. NB-IoT provided up to 22.8 kbps in the downlink direction. Table 4 shows that the corresponding throughput of 23.0 kbps was in line with theoretical calculations and simulations. In the uplink direction, the evaluation found a maximum goodput of 22.4 kbps and a throughput of 22.6 kbps. This value was significantly below the theoretical maximum of 62.5 kbps, which could be due to device limitations. However, both uplink and downlink throughput found in this evaluation significantly exceeded the values of previous measurements [11]. In both directions, the data rates were stable over a wide range of signal levels, before decreasing at an RSRP signal level of −120 dBm. Since the installation location of a IoT device can not be freely chosen in most cases, this increases installation flexibility. In extreme coverage situations, the 3GPP specifies a minimum throughput of 160 bps at 164 dB MCL, which was confirmed in the simulations [1], but could only be approximated in the evaluation at 160 dB MCL. This aspect will be subject to further investigations in the future.

## 6. Conclusions

In this paper, a detailed evaluation of the end-user QoS of the NB-IoT LPWAN standard was presented. One of the first systematic analyses of the relevant physical and application layer QoS parameters was conducted in a real network. Moreover, the influence of these parameters on the end-user QoS was studied in the uplink and downlink direction.

Different experiments were conducted to verify the performance of NB-IoT in a public network using commercially available hardware. Analyzing the NB-IoT coverage showed that the 3GPP target MCL of 164 dB [4] was closely approximated, but could be reduced by external interference. The necessary high receiver sensitivity was achieved by using discrete levels of signal repetitions, which were referred to as ECL. Using the highest repetition level ECL2, signals below the noise floor could be detected, which allowed sensor installations under difficult conditions.

On the other hand, the number of ECL repetitions was a tradeoff between system latency and coverage. In the downlink direction, NB-IoT maintained a latency below one second in all coverage scenarios. In the uplink, NB-IoT provided low latency for ECL0 and ECL1 transmissions. However, when ECL2 was used below the noise floor, the latency increased to tens of seconds. This behavior could be attributed to the high number of signal repetitions and the allocation of individual subcarriers over a longer duration. Configuring the modem to send exception messages as specified by the 3GPP requirements did not improve the latency, which might be because the mobile network did not yet implement this functionality.

NB-IoT provided a consistent data rate over a wide range of signal levels. In the downlink direction, the throughput of 23.0 kbps was within the expected range. However, in the uplink, the data rate depended on the allocation scheme. The obtained throughput of 22.6 kbps was significantly lower than the theoretical maximum for a transmission using 12 subcarriers, which requires further investigation in a future work.

Overall, NB-IoT satisfied most of the theoretical 3GPP QoS requirements [4] in a commercial deployment and was a suitable technology for a wide range of sensor applications. The QoS might be reduced for signal levels below the noise floor, which must be taken into consideration during application development. In the case of smart metering, four transmission categories were analyzed, and NB-IoT was verified to be appropriate if latency values above 10 s were acceptable for signal levels below the noise floor.

In future research, NB-IoT will be compared to other LPWAN technologies in the context of smart metering use cases. This analysis will include the currently unavailable NB-IoT non-IP optimizations, as well as the 3GPP Release 14 improvements.

## Figures and Tables

**Table 1:** NB-IoT exception report latency (adapted from [1]).

Coupling Loss [dB]	Stand-Alone Mode Latency [s]	Guard-Band Mode Latency [s]	In-Band Mode Latency [s]
144	0.3	0.3	0.3
154	0.7	0.9	1.1
164	5.1	8.0	8.3

**Table 2:** Reference link budget configuration achieving the minimum requirement of MCL = 164 dB for 5G mMTC systems as defined by IMT-2020 [19] with 32 and 128 repetitions in uplink and downlink (adapted from [6]).

	NPUSCH 164 dB MCL Ref. Performance	NPDSCH 164 dB MCL Ref. Performance
Transmit Power $P_{TX}$ (dBm)	23	35
TBS (Bit)	1000	680
Repetitions $N_{Rep}$	32 (max. 128)	128 (max. 2048)
Resource Units $N_{RU}$	10	8
BLER (%)	10	10
$SNR_{min}$ (dB)	−13.8	−14.7
Noise Figure NF (dB)	5	7
Interference Margin (IM) (dB) ^1^ (1)	0	0
Sensitivity $P_{Rx,min}$ (dBm)	−141	−129
MCL (dB)	164	164

^1^ In mobile networks, additional interference degrades the MCL, especially in dense urban areas.

**Table 3:** Mean values ($\mu_{RSRP}$, $\mu_{RSSI}$) and standard deviation ($\sigma_{RSRP}$, $\sigma_{RSSI}$) of the RSRP and RSSI measurements.

Attenuation [dB]	Number of Measurements	$\mu_{\mathrm{RSRP}}$ [dBm]	$\sigma_{\mathrm{RSRP}}$ [dB]	$\mu_{\mathrm{RSSI}}$ [dBm]	$\sigma_{\mathrm{RSSI}}$ [dB]
10	30	−98.052	1.406	−90.237	0.909
15	30	−102.325	0.720	−94.827	0.592
20	30	−108.063	1.183	−99.966	0.631
25	30	−113.352	1.163	−104.787	0.680
30	30	−118.548	1.647	−108.580	0.647
35	30	−121.862	1.513	−110.743	0.429
40	30	−125.244	1.513	−110.834	0.293
45	30	−129.202	1.009	−111.768	0.613
50	30	−133.635	1.716	−112.210	0.932

**Table 4:** Comparison of different NB-IoT QoS parameters in a guard-band deployment.

Metric	3GPP Specification [4]	Simulated Performance [1]	Measured Performance
MCL (dB)	164	164	163.5
Max. uplink throughput (kbps)	62.5 ^1^	62.6 ^1^	22.6 ^2^
Max. downlink throughput (kbps)	27.2 ^1^	26.2 ^1^	23.0
Min. data rate (kbps) (164 dB MCL)	0.160	UL: 0.320 DL: 0.370	>0.707 @ 160 dB MCL
Exception report latency (s) (164 dB MCL)	10	8.0	UL: 11.2, DL: 0.610 for user traffic

^1^ The specification and simulation assume 180 kHz multi-tone transmissions for maximum throughput.

^2^ The uplink data rate depends on the allocation scheme and the number of subcarriers used.

## Abbreviations

The following abbreviations are used in this manuscript:

3GPP	Third Generation Partnership Project
4G	Fourth Generation of Mobile Networks
5G	Fifth Generation of Mobile Networks
*A*	Antenna Gain
ACK	Acknowledgment
ASCII	American Standard Code for Information Interchange (encoding)
*B*	Bandwidth
BLER	Block Error Rate
BPSK	Binary Phase Shift Keying
cMTC	Critical Machine Type Communication
CL	Coupling Loss
DL	Downlink
ECL	Enhanced Coverage Level
eNodeB	Evolved Node B (base station in a LTE network)
GSM	Global System for Mobile Communication
HARQ	Hybrid Automatic Repeat Request
IM	Interference Margin
IMT-2020	International Mobile Telecommunications 2020 Specification
IoT	Internet of Things
IP (-v4, -v6)	Internet Protocol (Version 4, Version 6)
ISM	Industrial, Scientific and Medical Frequency Band
*k*	Boltzmann’s constant
LoRaWAN	Long-Range Wide Area Network (LPWAN technology)
LPWAN	Low-Power Wide Area Network
LTE	Long-Term Evolution
LTE Cat-M1	LTE Category M1 (LPWAN technology)
μ	Mean value
MAC	Medium Access Control
MCL	Maximum Coupling Loss
MCS	Modulation and Coding Scheme
mMTC	Massive Machine Type Communication
MNO	Mobile Network Operator
MTC	Machine Type Communication
*N*	Number of measurement samples
$N_{Rep}$	Number of ECL repetitions
$N_{RU}$	Number of resource units
$N_{SF}$	Number of subframes
NACK	Negative Acknowledgment
NB-IoT	Narrowband Internet of Things
NF	Noise Figure
NLOS	Non-Line of Sight
NPDCCH	Narrowband Physical Downlink Control Channel
NPDSCH	Narrowband Physical Downlink Shared Channel
NPUSCH	Narrowband Physical Uplink Shared Channel
NRS	Narrowband Reference Symbol
NTP	Network Time Protocol
OFDM	Orthogonal Frequency Division Multiplexing
$P_{CMAX,c(i)}$	Cell-specific maximum transmit power on slot i
$P_{I}$	Interference power
$P_{N}$	Noise power
$P_{N,eff}$	Effective noise power
$P_{RX,min}$	Sensitivity
$P_{TX}$	Transmission power at the antenna connector
PDCP	Packet Data Convergence Protocol
PHY	Physical Layer
PRB	Physical Resource Block
QoS	Quality of Service
QPSK	Quadrature Phase Shift Keying
$R_{max}$	Maximum data rate
RB	Resource Block
RE	Resource Element
RF	Radio Frequency
Rel-13/14/15	3GPP LTE Release Version 13/14/15
RLC	Radio Link Control
ROHC	Robust Header Compression
RSRP	Reference Signal Received Power
RSRQ	Reference Signal Received Quality
RSSI	Received Signal Strength Indicator
RU	Resource Unit
RX	Receiver
σ	Standard deviation
SC-FDMA	Single Carrier Frequency-Division Multiple Access
SF	Subframe
SINR	Signal-to-Interference and Noise Power
SMS	Short Message Service
SNR	Signal-to-Noise Ratio
SRD	Short Range Devices Frequency Band
*T*	Temperature
$T_{min}$	Minimum transmission time
$T_{RU}$	Transmission time of one resource unit
$T_{SF}$	Transmission time of one subframe
TBS	Transport Block Size
TX	Transmitter
UDP	User Datagram Protocol
UE	User Equipment (mobile network modem)
UL	Uplink
USB	Universal Serial Bus
WSN	Wireless Sensor Networks
*x*	Subcarrier activity factor
